# Prevention Measures for COVID-19 and Changes in Kawasaki Disease Incidence

**DOI:** 10.2188/jea.JE20210132

**Published:** 2021-11-05

**Authors:** Nobuyuki Katsumata, Daisuke Harama, Takako Toda, Yuto Sunaga, Masashi Yoshizawa, Yosuke Kono, Yohei Hasebe, Keiichi Koizumi, Minako Hoshiai, Tomohiro Saito, Sho Hokibara, Koji Kobayashi, Miwa Goto, Tomoaki Sano, Makoto Tsuruta, Makoto Nakamura, Sonoko Mizorogi, Masanori Ohta, Mie Mochizuki, Hiroki Sato, Hiroshi Yokomichi, Takeshi Inukai

**Affiliations:** 1 Department of Pediatrics, University of Yamanashi, Yamanashi, Japan; 2 Departments of Neonatology, Yamanashi Central Prefectural Hospital, Yamanashi, Japan; 3 Department of Pediatrics, Yamanashi Central Prefectural Hospital, Yamanashi, Japan; 4 Department of Pediatrics, Fujiyoshida Municipal Hospital, Yamanashi, Japan; 5 Department of Pediatrics, Kofu Municipal Hospital, Kofu, Yamanashi, Japan; 6 Department of Pediatrics, Yamanashi Kosei Hospital, Yamanashi, Japan; 7 Department of Pediatrics, National Hospital Organization Kofu National Hospital, Yamanashi, Japan; 8 Department of Pediatrics, Yamanashi Red Cross Hospital, Yamanashi, Japan; 9 Department of Pediatrics, Kofu-kyoritsu Hospital, Yamanashi, Japan; 10 Department of Pediatrics, Nirasaki City Hospital, Yamanashi, Japan; 11 Department of Pediatrics, Tsuru Municipal General Hospital, Yamanashi, Japan; 12 Department of Pediatrics, Kyonan Medical Center Fujikawa Hospital, Yamanashi, Japan; 13 Department of Pediatrics, Suwa Central Hospital, Nagano, Japan; 14 Department of Health Sciences, University of Yamanashi, Yamanashi, Japan

**Keywords:** COVID-19, Kawasaki disease, retrospective database, infectious disease

## Abstract

**Background:**

Kawasaki disease is suspected to be triggered by previous infection. The prevention measures for coronavirus disease 2019 (COVID-19) have reportedly reduced transmission of certain infectious diseases. Under these circumstances, the prevention measures for COVID-19 may reduce the incidence of Kawasaki disease.

**Methods:**

We conducted a retrospective study using registration datasets of patients with Kawasaki disease who were diagnosed in all 11 inpatient pediatric facilities in Yamanashi Prefecture. The eligible cases were 595 cases that were diagnosed before the COVID-19 pandemic (from January 2015 through February 2020) and 38 cases that were diagnosed during the COVID-19 pandemic (from March through November 2020). Incidence of several infectious disease were evaluated using data from the Infectious Disease Weekly Report conducted by the National Institute of Infectious Diseases.

**Results:**

Epidemics of various infectious diseases generally remained at low levels during the first 9 months (March through November 2020) of the COVID-19 pandemic. Moreover, the incidence of COVID-19 was 50–80 times lower than the incidence in European countries and the United States. The total number of 38 cases with Kawasaki disease for the 9 months during the COVID-19 pandemic was 46.3% (−3.5 standard deviations [SDs] of the average [82.0; SD, 12.7 cases] for the corresponding 9 months of the previous 5 years. None of the 38 cases was determined to be triggered by COVID-19 based on their medical histories and negative results of severe acute respiratory syndrome coronavirus 2 testing at admission.

**Conclusion:**

These observations provide a new epidemiological evidence for the notion that Kawasaki disease is triggered by major infectious diseases in children.

## INTRODUCTION

Coronavirus disease 2019 (COVID-19), caused by severe acute respiratory syndrome coronavirus 2 (SARS-CoV-2), spread rapidly around the world. During the COVID-19 pandemic, substantial reductions have been observed in the transmission of seasonal respiratory viruses, particularly influenza and respiratory syncytial virus (RSV).^1^^–^^4^ Reductions in the person-to-person transmission of viruses may be attributed to the community-level prevention measures for COVID-19, such as wearing face masks, washing hands frequently, and staying at home (eg, school closures and the promotion of remote working).^5^^,^^6^

Kawasaki disease is an acute systemic vasculitis of unknown cause that predominantly affects infants and young children.^7^^,^^8^ To date, although the direct cause initiating Kawasaki disease remains unknown, it is suspected to be triggered by some infectious diseases.^9^^,^^10^ However, it has been not epidemiologically elucidated whether prevention measures for infectious diseases decrease the incidence of Kawasaki disease in children or not. Meanwhile, during the COVID-19 pandemic in Europe and the United States, the emergence of a hyperinflammatory syndrome similar to Kawasaki disease was noted among children, several days after SARS-CoV-2 infection.^11^^–^^16^ Before the COVID-19 pandemic, Kawasaki disease was more common in East Asian countries, particularly Japan.^17^^,^^18^ In contrast, the prevalence of COVID-19 has tended to be mild in East Asian countries, including Japan.^19^^,^^20^ Under these circumstances, during the COVID-19 pandemic, the incidence of Kawasaki disease not triggered by COVID-19 in Japan may have decreased as a result of a possible decline in the transmission of infectious diseases due to COVID-19 prevention measures. In this report, we describe the significant reduction in cases of Kawasaki disease during the first 9 months of the COVID-19 pandemic in one of the 47 Japanese prefectures.

## METHODS

We conducted a retrospective review of registration datasets of patients diagnosed in Yamanashi Prefecture. Ethical approval of the study was given by the Research Ethics Committee of the University of Yamanashi Hospital (approved on September 27, 2017; Approval Number 1698). The study is a multicenter analysis of patients under the 15 years of age who were diagnosed in all 11 inpatient facilities caring for pediatric patients in Yamanashi Prefecture. This is the only population-based prefectural registration database of Kawasaki disease in Japan. At the end of every year, anonymized clinical records of all the annually diagnosed cases at each facility were retrospectively collected. As a result, all of the patients diagnosed in the facilities in Yamanashi were enrolled in the study. The incidence rate was compensated by annual demographics data published by the Yamanashi Prefectural Government. A comparison of epidemiologic features was conducted between 1,175 cases enrolled in the Yamanashi study over 12 years, from 2007 through 2018, and 169,556 cases enrolled in a nationwide epidemiologic survey over the same 12 years.^21^^–^^25^ In the nationwide survey, a questionnaire was biyearly sent to pediatric departments in hospitals with more than 100 beds (1,881 facilities in the 2017 survey) throughout Japan. The response rate to the survey conducted in 2017 was reported to be 77%.^25^ The nationwide survey in the 12 years included 895 cases (76%) of the 1,175 cases reported by the facilities in Yamanashi. Comparisons of monthly and seasonal (3-month) incidence and clinical characteristics of Kawasaki disease in Yamanashi were conducted between 38 cases diagnosed from March through November 2020 (during the COVID-19 pandemic) and 595 cases diagnosed from January 2015 through February 2020 (before the COVID-19 pandemic).

### Diagnosis and treatment of Kawasaki disease

Diagnosis of Kawasaki disease was retrospectively confirmed based on criteria defined in the sixth edition of the Japanese Kawasaki disease diagnostic guidelines.^26^ In brief, diagnosis was made for patients exhibiting at least five of the six major symptoms, or patients with coronary artery lesions even if they had only four of the six major symptoms. The patients were initially administered 2 g/kg of intravenous immunoglobulin (IVIG) and 30 mg/kg of oral aspirin. Response to the initial treatment was evaluated as effective if the body temperature was less than 37.5°C and the serum C-reactive protein (CRP) level was less than half of the peak value within 48 hours after IVIG administration. Patients with initial IVIG resistance were treated to an additional 2 g/kg of IVIG or intravenously 5 mg/kg infliximab (IFX).^27^ For those patients that exhibited resistance to either additional IVIG or IFX therapy, plasma exchange was subsequently carried out.^28^

### COVID-19 and other infectious disease surveillance

COVID-19 epidemic data for Yamanashi and for Japan were extracted from the open access datasets maintained by the Yamanashi Prefectural Government^29^ and by Japan’s Ministry of Health, Labour and Welfare,^30^ respectively. Data on representative infectious diseases in Yamanashi and in Japan were compared between the 2020 season and the previous seven seasons (2013–19) using data from the Infectious Disease Weekly Report (IDWR) conducted by the National Institute of Infectious Diseases (NIID).^31^ The IDWR data were collected every week from approximately 5,000 sentinel centers (about 60% of which were pediatrics) across Japan, including hospitals and clinics. Diagnosis of each infectious disease was made based on clinical symptoms and/or laboratory findings.

### Statistical analysis

All statistical analyses were performed with EZR (version 1.40; Saitama Medical Center, Jichi Medical University, Saitama, Japan), which is a graphical user interface for R (The R Foundation for Statistical Computing, Vienna, Austria). To assess trends in the monthly and seasonal changes of Kawasaki disease, we applied a statistical process control (SPC) chart.^32^ In the analysis of monthly and seasonal changes, mean and standard deviation (SD) were calculated from data for 2015–19. Reductions are considered to be significant when: (1) any one point is less than 3 SD from the mean; (2) two out of three consecutive points are less than 2 SD; and (3) four out of five consecutive points are less than 1 SD.^32^ The Mann–Whitney U test was performed to compare monthly and seasonal incidences between the 2020 season and the 2015–19 seasons.

## RESULTS

### Epidemiologic feature of Kawasaki disease in Yamanashi and Japan

**Figure 1.**
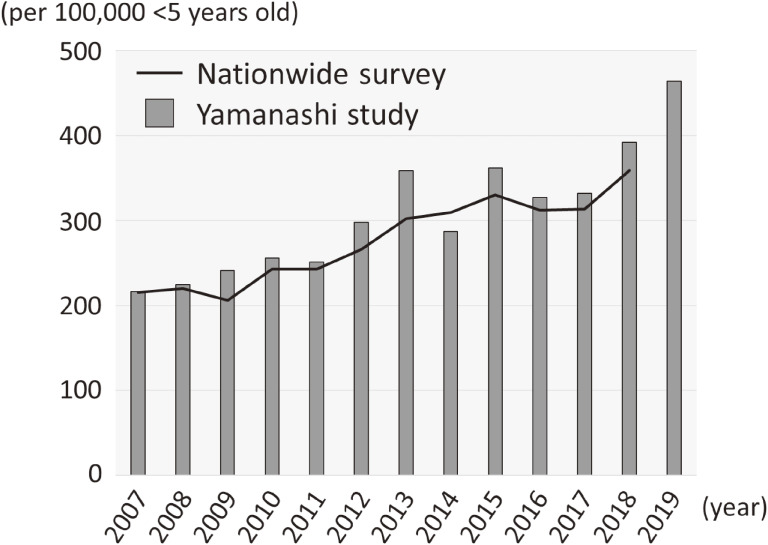
Epidemiologic features of Kawasaki disease in Yamanashi and Japan. The bars indicate the incidence rate (per 100,000 children aged 0–4 years/year) in the Yamanashi study (2007–19), and the solid line indicates that in the nationwide survey (2007–18). Nationwide data for 2019 were unavailable at the time of manuscript preparation (January 2021).

Yamanashi Prefecture is located to the west of the Tokyo metropolitan area, and its population is 0.811 million (in 2018). In the 12 years from 2007 through 2018, 1,175 newly diagnosed cases of Kawasaki disease were enrolled in the Yamanashi study. In a nationwide epidemiologic survey,^21^^–^^25^ 169,556 cases were enrolled in the same 12 years. Male predominance was observed both in the Yamanashi study (646/1,175, 55.0%) and in the nationwide survey (97,280/169,556; 57.4%). Almost 90% of cases were of patients less than 5 years old, in both the Yamanashi study (1,036/1,175, 88.2%) and the nationwide survey (147,963/169,556, 87.3%). Annual changes in the incidence rate of the Yamanashi study showed a similar increasing trend to that observed in the nationwide survey (Figure 1). The incidence rates in 2007 and 2018 were 217 and 392 cases per 100,000 children aged 0–4 years, respectively, in the Yamanashi study, and 215 and 359 cases per 100,000 children aged 0–4 years, respectively, in the nationwide survey. In every year except 2014, the incidence rate in the Yamanashi study was slightly higher than that in the nationwide survey. These observations demonstrate that the epidemiologic features of Kawasaki disease in the Yamanashi study may represent a nationwide trend in Japan.

### Epidemics of COVID-19 in Yamanashi and Japan

**Figure 2.**
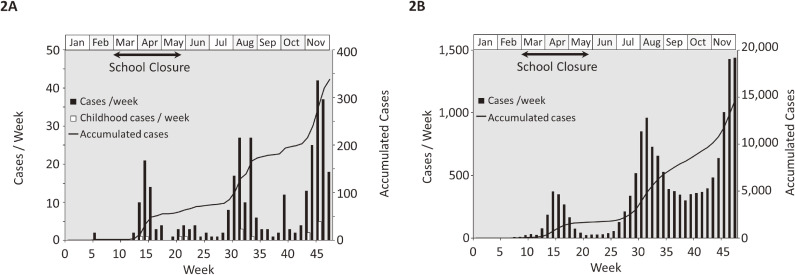
Epidemics of COVID-19 in Yamanashi **(A)** and Japan **(B)**. The horizontal axis shows calendar weeks in 2020. The closed bars (left vertical axis) show numbers of weekly diagnosed cases. The solid lines (right vertical axis) show the cumulative number of cases. The open bars show numbers of pediatric (aged less than 14 years) cases in Yamanashi. COVID-19, coronavirus disease 2019.

The first COVID-19 case in Yamanashi was identified in calendar week 6 of 2020 (Figure 2A). On March 2 (week 10), the Japanese government announced nationwide school closures in its intensified COVID-19 response. School closures continued until May 31 (week 22). Both in Yamanashi (Figure 2A) and in Japan (Figure 2B), three epidemic peaks were observed before the end of November 2020 (week 48); weeks 13–19, 27–35, and from week 43. By week 48, 388 cases had been diagnosed in Yamanashi. Among those, 13 cases were pediatric cases, but none of them developed a multisystem inflammatory syndrome,^14^^–^^16^ including Kawasaki-like disease. The incidences of COVID-19 cases by week 48 in Yamanashi^29^ and Japan^30^ were 48 and 116 per 100,000 population, respectively; approximately 50–80 times lower than those in the United States (4,158) and European countries (France, 3,421; Italy, 2,642; the United Kingdom, 2,427).^19^ These observations indicate that the prevalence of COVID-19 was mild in Yamanashi up to the end of November 2020.

### Epidemics of infectious diseases in 2020 in Yamanashi and Japan

**Figure 3.**
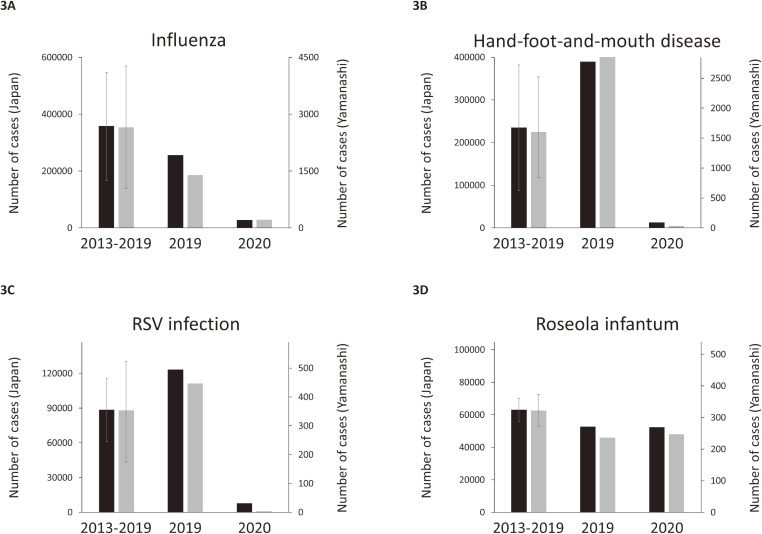
Epidemics of infectious diseases in 2020 season. Epidemics of influenza **(A)**, hand-foot-and-mouth disease **(B)**, RSV infection **(C)**, and roseola infantum **(D)** in Yamanashi and Japan, based on the Infectious Disease Weekly Report (IDWR) conducted by the National Institute of Infectious Diseases (NIID). The vertical axes show the cumulative numbers of cases for the 9 months from March through November reported by sentinel centers in Japan (left axis) and Yamanashi (right axis). In each panel, the left pair of bars with ranges show average numbers with SD in the previous 7 years (2013–19), while the middle and right pairs of bars show the cumulative numbers of cases in the 2019 and 2020, respectively. Black bars: Japan. Gray bars: Yamanashi. RSV, respiratory syncytial virus; SD, standard deviation.

We compared epidemics of representative infectious diseases in Yamanashi and Japan between the 2020 season and the previous seven seasons (2013–19), using the IDWR database conducted by the NIID^31^ (Figure 3). Incidence of influenza declined in February 2020 and remained at a low level (eFigure 1 and eFigure 2). Accumulated numbers of influenza cases for the 9 months of 2020 (March through November) in Yamanashi and Japan were 8.0% (215 vs 2,656 [SD, 1,608]) and 7.8% (27,990 vs 357,912 [SD, 189,516]) of the averages for the same 9 months of the previous 7 years, respectively (Figure 3A). Similar patterns of decline were observed in the incidence of infectious gastroenteritis, mycoplasma pneumonia, and streptococcal pharyngitis in Yamanashi and in Japan. The accumulated numbers of cases of infectious gastroenteritis, mycoplasma pneumonia, and streptococcal pharyngitis in Yamanashi for the 9 months of 2020 were 36%, 28%, and 27% of the averages, respectively (eFigure 3A, eFigure 3B, and eFigure 3C). No summer outbreaks of herpangina or hand-foot-and-mouth disease, or autumn outbreaks of RSV infection, were observed in 2020, either in Yamanashi or in Japan. The cumulative numbers of cases of hand-foot-and-mouth disease and RSV infection in Yamanashi for the 9 months of 2020 were 2.2%, and 1.1% of the averages, respectively (Figure 3B and Figure 3C). In contrast, the activity of roseola infantum (exanthema subitem) for the 9 months of 2020 was almost comparable to the previous seasons (Figure 3D). These observations demonstrate that epidemics of various infectious diseases (except for roseola infantum) generally remained at low levels in Yamanashi and Japan during the first 9 months of the COVID-19 pandemic.

### Epidemiologic feature of Kawasaki disease in 2020 in Yamanashi

**Figure 4.**
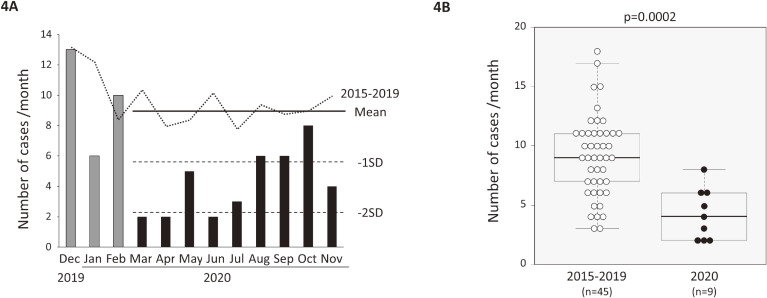
Epidemiologic features of Kawasaki disease in 2020 in Yamanashi. **(A)** Statistical process control chart of monthly changes in the number of cases. The dotted sequential line shows the average of monthly cases in the previous 5 years (2015–19). The closed bars indicate the number of monthly cases from December 2019 through November 2020. The solid and dotted straight lines indicate the mean, −1 SD, and −2 SD of monthly cases in the previous 5 years, respectively. **(B)** Bar plots comparing monthly cases between 9 months (March through November) of 2020 (*n* = 9) and the corresponding 9 months of the previous 5 years (*n* = 45). *P*-values in the Mann–Whitney U test are indicated. SD, standard deviation.

First, we simply compared the average number of cases in each month of 2015–2019 with the number of cases in each month of 2020. Among 11 months from January through November 2020, a significant reduction in student’s *t*-test was observed in 6 months (March, April, May, June, August, and November) (Table 1). We next compared the incidence of Kawasaki disease between 2020 and the previous 5 years (2015–19) using the SPC chart^32^ to evaluate the number of cases enrolled in the Yamanashi study. In January and February 2020, six and 10 cases were diagnosed, respectively. This was almost comparable to the average number of monthly cases (mean 10.3; SD, 4.1 cases) in January and February of the previous 5 years (Figure 4A). For the 9 months from March through November in 2015–19, the average number of monthly cases was 9.1 (SD, 3.5). In March, April, and May 2020, which was the school closure period, 2 (−2.0 SD), 2 (−2.0 SD), and 5 (−1.2 SD) cases were diagnosed, respectively. This reduction was statistically significant, since a reduction of greater than −2 SD was confirmed in two out of three consecutive points.^32^ In June and July 2020, after schools reopened, 2 (−2.0 SD) and 3 (−1.7 SD) cases were diagnosed, respectively. Thus, in 5 consecutive months (March through July 2020), a significant reduction (less than −1 SD in five or four out of five consecutive points)^32^ was confirmed. Subsequently, in August, September, October, and November 2020, 6 (−0.9 SD), 6 (−0.9 SD), 8 (−0.3 SD), and 4 (−1.5 SD) cases were diagnosed, respectively. The numbers of monthly cases (median, 4 cases) for the 9 months of 2020 (March through November) were significantly smaller than for the corresponding 9 months of the previous 5 years (9 cases) (*P* = 0.0002 in the Mann–Whitney U test) (Figure 4B). The same pattern was observed in the incidence rate, which was compensated by annual demographics (eFigure 4A and eFigure 4B). In 5 consecutive months (March through July 2020), a significant reduction (less than −1 SD in five or four out of five consecutive points)^32^ was confirmed.

**Table 1.** The number of cases for each month in 2015–2019 with those for the same month in 2020

	Jan	Feb	Mar	Apr	May	Jun	Jul	Aug	Sep	Oct	Nov
Before COVID-19 pandemic^a^											
Incidence (SD)	38.8 (15.8)	26.6 (7.5)	33.2 (14.1)	25.3 (10.2)	27.1 (6.4)	33.4 (20.8)	25.4 (15.5)	30.3 (6.7)	28.5 (11.5)	29.3 (11.8)	32.4 (4.8)
During COVID-19 pandemic^b^											
Incidence	20.2	33.7	6.7	6.7	17.3	6.9	10.4	20.8	20.8	27.8	13.9
*P* value^c^	0.057	0.104	0.014	0.016	0.026	0.046	0.096	0.033	0.21	0.784	0.001

COVID-19, coronavirus disease 2019; SD, standard deviation.

^a^Diagnosed from January 2015 through February 2020.

^b^Diagnosed from March 2020 through November 2020.

^c^Student’s *t*-test.

Next, we compared seasonal (3-month) changes (eFigure 5A). In the preceding winter season (December 2019 through February 2020), 29 cases were diagnosed. This was almost comparable to the average (33.0; SD, 3.4 cases) in the winters of the previous 5 years. The average number of cases in spring (March through May), summer (June through August), and autumn (September through November) of the previous 5 years was 27.3 (SD, 5.4). In the spring season of 2020, which was the school closure period, 9 (−3.4 SD) cases were diagnosed. In the following summer and autumn seasons of 2020, 11 (−3.0 SD) and 18 (−1.7 SD) cases were diagnosed, respectively. The incidence remained at a significantly low level for three consecutive seasons of 2020, since a reduction of less than −2 SD was confirmed in two out of three consecutive points.^32^ The numbers of seasonal cases (median, 11 cases) in the three seasons during the COVID-19 pandemic were significantly smaller than in the same three seasons of the previous 5 years (29 cases) (*P* = 0.011 in the Mann-Whitney U test) (eFigure 5B). The same pattern was observed in the incidence rate (eFigure 5C and eFigure 5D). In two out of three consecutive seasons, a significant reduction (less than −2 SD in two out of three consecutive points)^32^ was confirmed.

Overall, 38 cases were diagnosed in the first 9 months of the COVID-19 pandemic. The average number of diagnosed cases in the same 9 months of the previous 5 years was 82.0 (SD, 12.7). Thus, the number of cases for the first 9 months of the COVID-19 pandemic was 46.3% (−3.5 SD) of the average. This was a statistically significant reduction, since it was less than −3 SD from the mean.^32^ These observations demonstrate that the incidence of Kawasaki disease in Yamanashi during the first 9 months of the COVID-19 pandemic remained at a significantly lower level than in the previous 5 years.

### Clinical characteristics of Kawasaki disease in 2020 in Yamanashi

We compared the clinical characteristics of Kawasaki disease between the 38 cases diagnosed during the COVID-19 pandemic (from March through November 2020) and the 595 cases diagnosed before the COVID-19 pandemic (from January 2015 through February 2020). Among the 38 cases, 35 underwent SARS-CoV-2 testing at admission (Polymerase chain reaction [PCR] analysis, 33 cases; antigen test, 2 cases). The exceptions were three cases diagnosed in March and April 2020. All test results were negative. No case had a history of direct contact with COVID-19 cases, and no outbreak of secondary COVID-19 infections occurred in each hospital. Thus, although we did not evaluate the titer of antibodies against SARS-CoV-2 at the post-acute phase, no case was considered to be Kawasaki disease triggered by COVID-19.

As indicated in Table 2, no significant differences were observed in treatment initiation date, as well as sex and age distributions, between the 38 cases diagnosed during the COVID-19 pandemic and the 595 cases diagnosed before it. The response rate to the IVIG treatment (71%) in the cases diagnosed during the COVID-19 pandemic was almost similar to that (78%) in the cases diagnosed before the COVID-19 pandemic. The prevalence of dilatation of the coronary artery (5.3%) in the cases diagnosed during the COVID-19 pandemic was similar to that (5.0%) in the cases diagnosed before the COVID-19 pandemic. These observations demonstrate that the clinical characteristics and therapeutic responses of the cases diagnosed in the first 9 months of the COVID-19 pandemic were similar to those diagnosed before it.

**Table 2.** Comparison of clinical features between 595 individuals diagnosed before COVID-19 pandemic and 38 individuals diagnosed during COVID-19 pandemic

Characteristics	Before COVID-19 pandemic^a^	During COVID-19 pandemic^b^	*P* values
Total individuals, *n*	595	38	
Age, months			
Mean (SE)	33 (1.0)	34 (6.2)	*P* = 0.25^c^
<12, *n* (%)	114 (19)	10 (26)	*P* = 0.29^d^
>60, *n* (%)	84 (14)	7 (18)	*P* = 0.47^d^
Male, %	56	61	*P* = 0.73^d^
Date of treatment initiation, median (SD)	5 (1.0)	5 (1.5)	*P* = 0.28^c^
IVIG response rate, %	78	71	*P* = 1.00^d^
Coronary artery dilatation, %	5.0	5.3	*P* = 1.00^d^

COVID-19, coronavirus disease 2019; IVIG, intravenous immunoglobulin; SD, standard deviation; SE, standard error.

^a^Diagnosed from January 2015 through February 2020.

^b^Diagnosed from March 2020 through November 2020.

^c^Mann–Whitney U test.

^d^Fisher’s exact test.

## DISCUSSION

Kawasaki disease is thought to be triggered by previous infection.^9^^,^^10^ A markedly increased incidence of Kawasaki or Kawasaki-like disease has been reported in an area extensively affected by the SARS-CoV-2.^11^^–^^16^ Thus, we evaluated the incidence of Kawasaki disease during the COVID-19 pandemic in Japan. In Yamanashi, during the first 9 months of the COVID-19 pandemic, incidence remained at significantly low levels compared to the previous 5 years. Since incidence of many infectious diseases was reduced (except for roseola infantum) during the COVID-19 pandemic, the substantial reduction in the incidence of Kawasaki disease in Yamanashi may reflect a reduction in the transmission of infectious diseases due to COVID-19 prevention measures. In this context, common age of onset in Kawasaki disease is similar to the peak ages of onset in common viral infections.^33^^–^^35^ Moreover, common respiratory viruses were frequently detectable in the airway specimens from patients with Kawasaki disease at diagnosis.^36^^,^^37^ Taken together our observations with these previous findings, reduced incidence of common infectious diseases in children due to the COVID-19 prevention measures may trigger the reduced incidence of Kawasaki disease.

Consistently, the most significant reduction in the incidence of Kawasaki disease was observed in the spring of 2020, during the school closure period. Although Kawasaki disease is commonly developed in younger children, we speculated that school closure may affect the reduced incidence of Kawasaki disease in younger children based on the following two observations. One is that outbreaks of acute infectious gastroenteritis and Streptococcal pharyngitis, both common infectious diseases among children (including schoolchildren) in the spring season, were well controlled during the school closure period in 2020. The other is the well-known epidemiological observation that influenza vaccination for schoolchildren reduced mortality from influenza among older persons in Japan.^38^

In January 2020, the number of the cases was almost half of the average for 2015–2019 with statistically marginal reduction (*P* = 0.057). However, this reduction in January 2020 was unlikely to be related to the COVID-19 pandemic based on the following two observations. One is that no significant reduction was observed the following February (2020). The other is that no practical COVID-19 prevention measures were started in January 2020 in Yamanashi, since no COVID-19 outbreak was observed.

Meanwhile, in the first 9 months of the COVID-19 pandemic, the incidence of COVID-19 in Yamanashi was 50–80 times lower than that in European countries and the United States. These observations suggest that the emergence of Kawasaki disease triggered by COVID-19 was very limited in Yamanashi and Japan, if it occurred at all.^39^ Indeed, no cases were considered to be Kawasaki disease triggered by COVID-19, based on medical histories and negative results of PCR analysis or antigen tests of SARS-CoV-2 on admission. As for COVID-19 prevention measures, there are two aspects: decreased social activity and appropriate personal hygiene. We speculate an involvement of both aspects in the decreased incidences of Kawasaki disease due to the following two reasons. First, in cellular phone location data during the COVID-19 pandemic,^40^ the most significant mobility reduction was observed in the first 3 months (March through May 2020), when the most significant reduction in the incidence of Kawasaki disease was observed. Next, in the summer season of 2020, significant reduction was observed in the incidence of summertime viral infections, such as hand-foot-and-mouth disease and herpangina, despite the fact that reduction in mobility was less significant in the cellular phone location data.

During the COVID-19 pandemic, it is speculated that numerous caregivers have avoided visiting hospitals to minimize the risk of SARS-CoV-2 infection.^5^^,^^6^^,^^41^ However, in the case of Kawasaki disease, high fever typically lasts for more than 5 days without specific treatment and is unlikely to recover spontaneously before visiting healthcare facilities. Thus, any effect of caregivers’ hesitation in approaching the healthcare system on the reduced incidence of Kawasaki disease was very small, if anything. In this context, there is a concern that delayed diagnosis of Kawasaki disease may be associated with poor therapeutic response or development of cardiac sequelae.^42^ However, we confirmed that both the date of treatment initiation and the response rate to the IVIG treatment, as well as the cardiac sequelae, in the cases occurring during the COVID-19 pandemic were almost comparable to those before it.

There are several limitations in this study. First, as it was a population-based study in a small geographical administrative unit, the number of participants was relatively small. Second, the reduced activities of infectious diseases other than COVID-19 may be overestimated. Since most of the infectious diseases are not serious, numerous caregivers of patients may have avoided visiting local sentinel centers, to minimize their risk of SARS-CoV-2 infection.

In conclusion, this study describes monthly and seasonal incidences of Kawasaki disease during the COVID-19 pandemic in Yamanashi Prefecture. It confirms that the incidence of Kawasaki disease was at significantly lower levels than before the COVID-19 pandemic. The incidences of both COVID-19 and other infectious diseases remained at lower levels. These findings suggest that the COVID-19 prevention measures reduce the incidence of Kawasaki disease in association with reduction of major infectious disease in children.

## APPENDIX A. SUPPLEMENTARY DATA

eFigure 1. Epidemics of infectious diseases in 2020 season in Yamanashi. Epidemics of influenza (A), infectious gastroenteritis (B), mycoplasma pneumonia (C), streptococcal pharyngitis (D), herpangina (E), hand-foot-and-mouth disease (F), RSV infection (G), and roseola infantum (H) in 2020 based on the Infectious Disease Weekly Report (IDWR) conducted by the National Institute of Infectious Diseases (NIID). The horizontal axis indicates months. The columns with error bars indicate the average and SD range of the previous 7 years (2013–19). The sequential line indicates cumulative number of cases in 2020.

eFigure 2. Epidemics of infectious diseases in 2020 season in Japan. Epidemics of influenza **(A)**, infectious gastroenteritis **(B)**, mycoplasma pneumonia **(C)**, streptococcal pharyngitis **(D)**, herpangina **(E)**, hand-foot-and-mouth disease **(F)**, RSV infection **(G)**, and roseola infantum **(H)** in 2020 based on the Infectious Disease Weekly Report (IDWR) conducted by the National Institute of Infectious Diseases (NIID). The horizontal axis indicates months. The columns with error bars indicate the average and SD range of the previous 7 years (2013–19). The sequential line indicates cumulative number of cases in 2020.

eFigure 3. Epidemics of infectious diseases. Epidemics of infectious gastroenteritis **(A)**, mycoplasma pneumonia **(B)**, streptococcal pharyngitis **(C)**, and herpangina **(D)** in Yamanashi and Japan, based on the Infectious Disease Weekly Report (IDWR) conducted by the National Institute of Infectious Diseases (NIID). The vertical axes show the cumulative numbers of cases for the 9 months from March to November reported by sentinel centers in Japan (left axis) and Yamanashi (right axis). In each panel, the left pair of bars with ranges show average numbers with SD in the previous 7 years (2013–19), while the middle and right pairs of bars show the cumulative numbers of cases in the 2019 and 2020, respectively. Black bars: Japan. Gray bars: Yamanashi.

eFigure 4. Epidemiologic features of Kawasaki disease in 2020 in Yamanashi (monthly). **(A)** Statistical process control chart of monthly changes in the incidence rate (per 100,000 <5 years old). The dotted sequential line indicates the average of monthly cases in the previous 5 years (2015–19). The closed bar indicates the incidence of monthly cases from December 2019 to November 2020. The solid and dotted straight lines indicate the mean, −1SD, and −2SD of monthly cases in the previous 5 years, respectively. **(B)** Bar plots comparing monthly incidence between 9 months (March to November) of 2020 (n = 9) and the corresponding 9 months of the previous 5 years. (n = 45). P-values in the Mann-Whitney U test are indicated.

eFigure 5. Epidemiologic features of Kawasaki disease in 2020 in Yamanashi (seasonal). **(A)** Statistical process control chart of seasonal changes in the number of cases. The dotted sequential line shows the average of seasonal cases from December 2019 to November 2020. The solid and dotted straight lines indicate the mean, −1 SD, −2 SD, and −3 SD of seasonal cases in the previous 5 years, respectively. **(B)** Bar plots comparing seasonal cases between three seasons (spring, summer, and autumn) of 2020 (n = 3), and the corresponding three seasons of the previous 5 years (n = 15). P-values in the Mann–Whitney U test are indicated. **(C)** Statistical process control chart of seasonal changes in the incidence rate (per 100,000 <5 years old). The dotted sequential line indicates the average of seasonal cases from December 2019 to November 2020. The solid and dotted straight lines indicate the mean, −1SD, −2SD, and −3SD of seasonal cases in the previous 5 years, respectively. **(D)** Bar plots comparing seasonal incidence between three seasons (spring, summer, and autumn) of 2020 (n = 3) and the corresponding three seasons of the previous 5 years (n = 15). P-values in the Mann-Whitney U test are indicated.
