# Supplementary material for: Prevention Measures for COVID-19 and Changes in Kawasaki Disease Incidence
Source: J Epidemiol. 2021 Nov 5;31(11):573–80. doi: 10.2188/jea.JE20210132 (PMC8502831; doi:10.2188/jea.JE20210132)
Supplement: Supplementary file 1 [file je-31-573-s001.pdf]

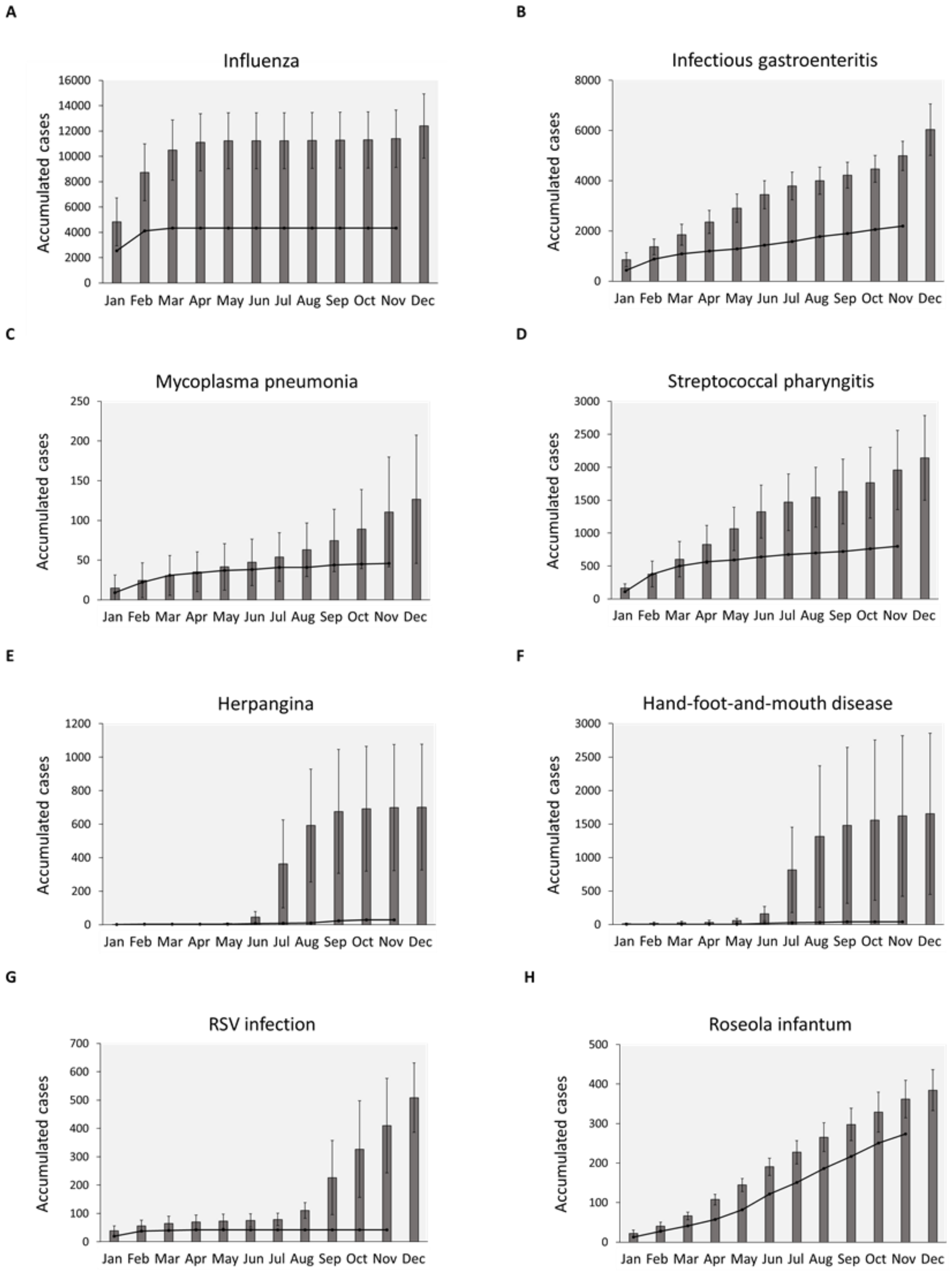

eFigure 1. Epidemics of infectious diseases in 2020 season in Yamanashi. Epidemics of influenza (A), infectious gastroenteritis (B), mycoplasma pneumonia (C), streptococcal pharyngitis (D), herpangina (E), hand-foot-and-mouth disease (F), RSV infection (G), and roseola infantum (H) in 2020 based on the Infectious Disease Weekly Report (IDWR) conducted by the National Institute of Infectious Diseases (NIID). The

horizontal axis indicates months. The columns with error bars indicate the average and SD range of the previous 7 years (2013–19). The sequential line indicates cumulative number of cases in 2020.

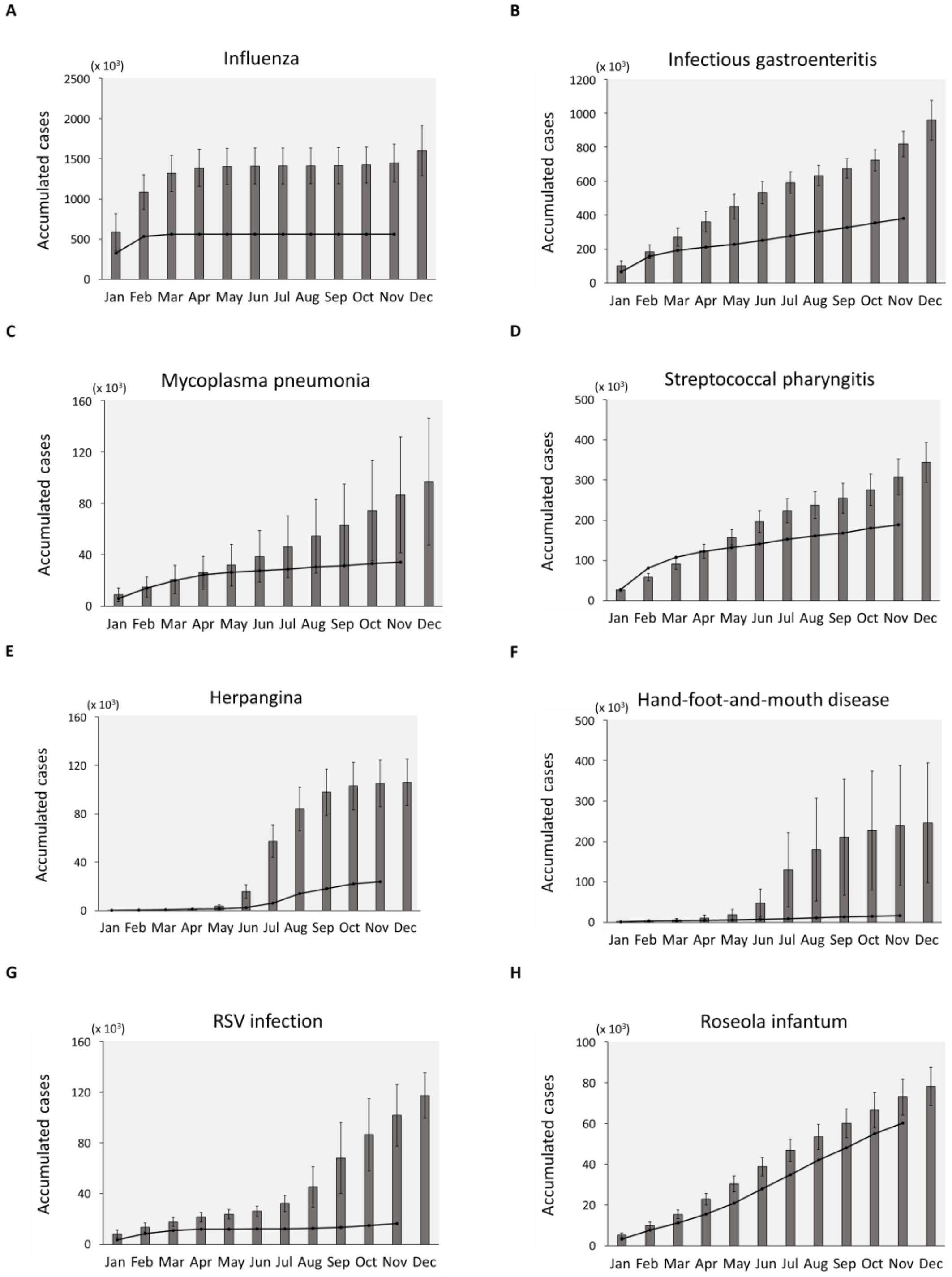

eFigure 2. Epidemics of infectious diseases in 2020 season in Japan. Epidemics of influenza **(A)**, infectious gastroenteritis **(B)**, mycoplasma pneumonia **(C)**, streptococcal pharyngitis **(D)**, herpangina **(E)**, hand-foot-and-mouth disease **(F)**, RSV infection **(G)**, and roseola infantum **(H)** in 2020 based on the Infectious Disease Weekly Report (IDWR) conducted by the National Institute of Infectious Diseases (NIID). The

horizontal axis indicates months. The columns with error bars indicate the average and SD range of the previous 7 years (2013–19). The sequential line indicates cumulative number of cases in 2020.

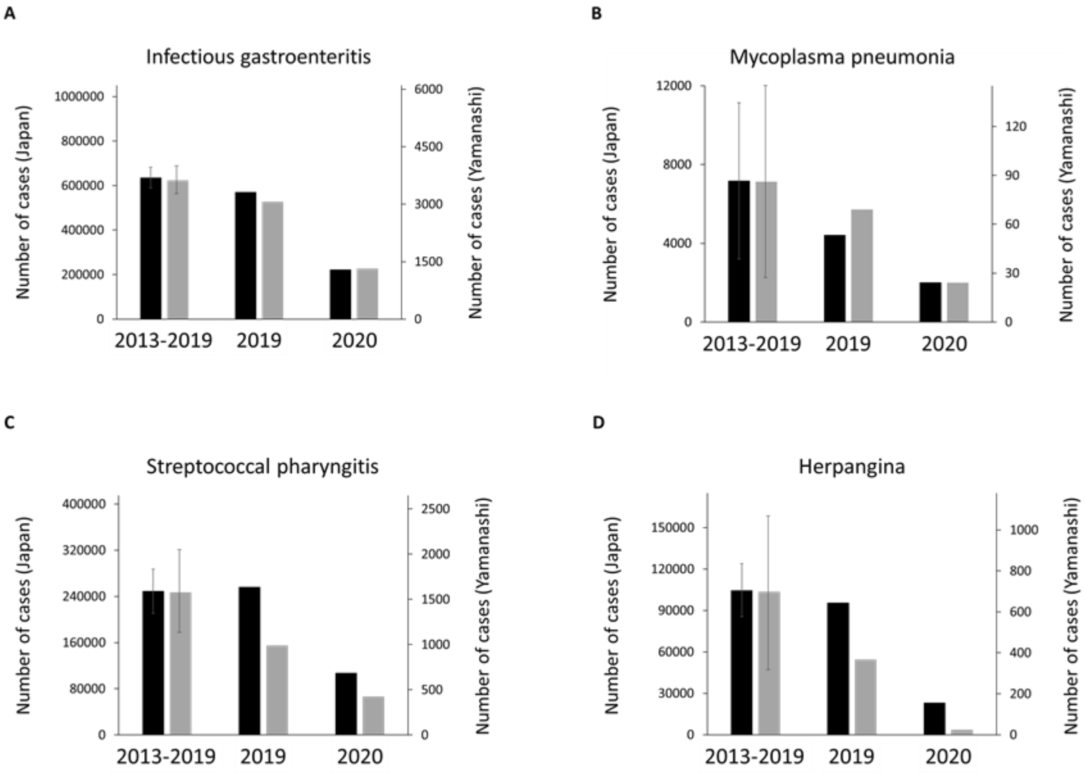

eFigure 3. Epidemics of infectious diseases. Epidemics of infectious gastroenteritis **(A)**, mycoplasma pneumonia **(B)**, streptococcal pharyngitis **(C)**, and herpangina **(D)** in Yamanashi and Japan, based on the Infectious Disease Weekly Report (IDWR) conducted by the National Institute of Infectious Diseases (NIID). The vertical axes show the cumulative numbers of cases for the 9 months from March to November reported by sentinel centers in Japan (left axis) and Yamanashi (right axis). In each panel, the left pair of bars with ranges show average numbers with SD in the previous 7 years (2013–19), while the middle and right pairs of bars show the cumulative numbers of cases in the 2019 and 2020, respectively. Black bars: Japan. Gray bars: Yamanashi.

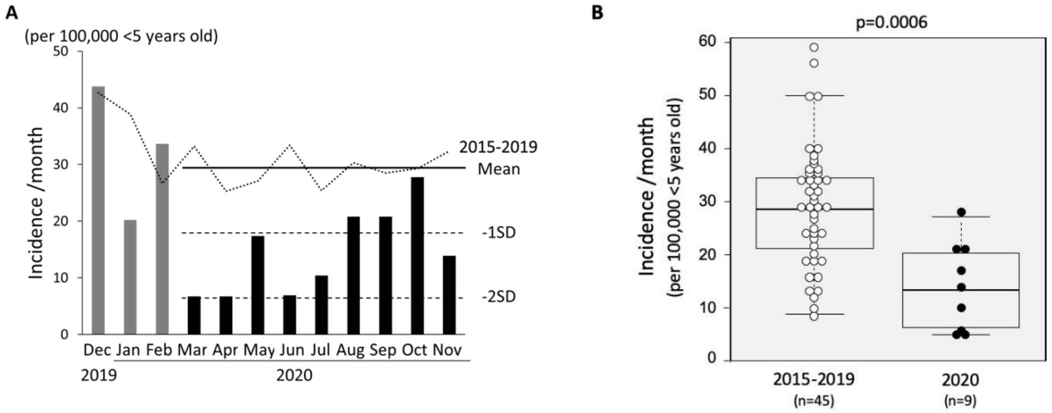

eFigure 4. Epidemiologic features of Kawasaki disease in 2020 in Yamanashi (monthly). **(A)** Statistical process control chart of monthly changes in the incidence rate (per 100,000 <5 years old). The dotted sequential line indicates the average of monthly cases in the previous 5 years (2015–19). The closed bar indicates the incidence of monthly cases from December 2019 to November 2020. The solid and dotted straight lines indicate the mean, -1SD, and -2SD of monthly cases in the previous 5 years, respectively. **(B)** Bar plots comparing monthly incidence between 9 months (March to November) of 2020 (n=9) and the corresponding 9 months of the previous 5 years. (n=45). P-values in the Mann-Whitney U test are indicated.

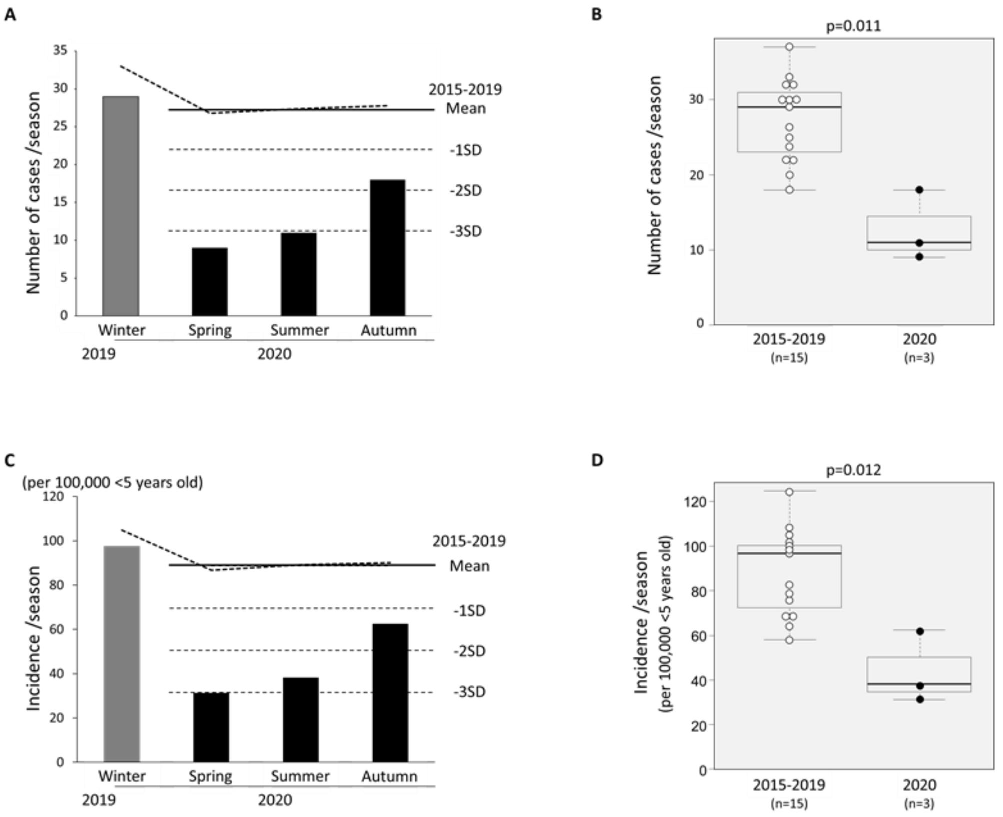

eFigure 5. Epidemiologic features of Kawasaki disease in 2020 in Yamanashi (seasonal). **(A)** Statistical process control chart of seasonal changes in the number of cases. The dotted sequential line shows the average of seasonal cases from December 2019 to November 2020. The solid and dotted straight lines indicate the mean, -1 SD, -2 SD, and -3 SD of seasonal cases in the previous 5 years, respectively. **(B)** Bar plots comparing seasonal cases between three seasons (spring, summer, and autumn) of 2020 (n=3), and the corresponding three seasons of the previous 5 years (n=15). P-values in the Mann–Whitney U test are indicated. **(C)** Statistical process control chart of seasonal changes in the incidence rate (per 100,000 <5 years old). The dotted sequential line indicates the average of seasonal cases from December 2019 to November 2020. The solid and dotted straight lines indicate the mean, -1SD, -2SD, and -3SD of seasonal cases in the previous 5 years, respectively. **(D)** Bar plots comparing seasonal incidence between three seasons (spring, summer, and autumn) of 2020 (n=3) and the corresponding three seasons of the previous 5 years (n=15). P-values in the Mann-Whitney U test are indicated.
